# Improved pentamethine cyanine nanosensors for optoacoustic imaging of pancreatic cancer

**DOI:** 10.1038/s41598-021-83658-3

**Published:** 2021-02-23

**Authors:** Matthew D. Laramie, Benjamin L. Fouts, William M. MacCuaig, Emmanuel Buabeng, Meredith A. Jones, Priyabrata Mukherjee, Bahareh Behkam, Lacey R. McNally, Maged Henary

**Affiliations:** 1 Department of Chemistry, Georgia State University, Atlanta, GA 30303 USA; 2 Department of Surgery, Oklahoma Health Science Center, Oklahoma City, 73104 USA; 3 Stephenson Cancer Center, Oklahoma Health Science Center, Oklahoma City, OK 73104 USA; 4 Department of Biomedical Engineering, University of Oklahoma, Norman, OK 72073 USA; 5 Center for Diagnostics and Therapeutics, Georgia State University, Atlanta, GA 30303 USA; 6 Department of Pathology, Oklahoma Health Science Center, Oklahoma City, OK 73104 USA; 7 Department of Mechanical Engineering, Virginia Tech University, Blacksburg, VA 24061 USA; 8 Department of Cancer Biology, Wake Forest University, Winston-Salem, NC 27157 USA

**Keywords:** Biological techniques, Cancer, Biomarkers, Diseases, Medical research, Chemistry

## Abstract

Optoacoustic imaging is a new biomedical imaging technology with clear benefits over traditional optical imaging and ultrasound. While the imaging technology has improved since its initial development, the creation of dedicated contrast agents for optoacoustic imaging has been stagnant. Current exploration of contrast agents has been limited to standard commercial dyes that have already been established in optical imaging applications. While some of these compounds have demonstrated utility in optoacoustic imaging, they are far from optimal and there is a need for contrast agents with tailored optoacoustic properties. The synthesis, encapsulation within tumor targeting silica nanoparticles and applications in in vivo tumor imaging of optoacoustic contrast agents are reported.

## Introduction

Diagnosis and treatment of pancreatic cancer remains daunting. Pancreatic tumors generally respond poorly to chemotherapy, and resection is the only truly curative option^1–7^. However, because pancreatic malignancies typically occur without symptoms, diagnosis is not usually made until the cancer has spread to other organs, at which point complete surgical resection is impossible^3,8,9^. Due to the combined difficulties of diagnosis and treatment, the prognosis for pancreatic cancer remains grim; 80–85% of patients will be untreatable at the time of diagnosis, and the overall 5-year survival rate is 4%^10^.

Numerous approaches to improve the detection of pancreatic lesions have been attempted, but little success has been achieved. Screening patients for a family history of pancreatic cancer may improve early detection, but clinical trials have not been performed to determine if this would result in any measurable improvement in patient outcomes^11–15^. Nevertheless, computed tomography (CT), magnetic resonance imaging (MRI) and endoscopic ultrasound (EUS) of patients with family histories of pancreatic cancers has shown promise in the detection of precancerous lesions^13^. EUS has shown the most accuracy in detecting small pancreatic neoplasia^12,13^. Unfortunately, due to poor resolution or sensitivity, microscopic lesions are still undetectable by current clinical imaging techniques^16,17^. One of the emerging technologies for medical imaging, optical imaging techniques, specifically fluorescence imaging, offer improved image resolution; however, the limited imaging depth reduces the clinical utility of fluorescence imaging for deep tissue imaging^18^.

Multispectral optoacoustic tomography (MSOT) combines the high sensitivity and resolution of optical imaging with the deeper imaging capabilities of ultrasound allowing for highly accurate imaging of microscopic anatomical features through several centimeters of tissue^19,20^. MSOT has shown promise in preclinical imaging tests for imaging tumors^21–25^, organ function^26,27^, and specific ions^28,29^. While MSOT can be used with endogenous chromophores (hemoglobin, and oxyhemoglobin), the highest imaging specificity can be achieved using exogenous contrast^30,31^. Particularly, contrast agents that absorb in the near infrared (NIR) window (650–900 nm) can be used to achieve exceptional signal-to-noise ratio (SNR) for in vivo imaging.

NIR chromophores for fluorescence imaging have been investigated, and dyes targeting specific tissues, targeting ligand-assisted dyes, and activatable fluorophores have been developed to image healthy and diseased tissues^32–37^. MSOT, on the other hand, is lacking in optimized dyes that can serve as contrast agents. The majority of the available literature utilizes commercially available NIR dyes, which have sub-optimal physicochemical properties and optoacoustic signal for in vivo use^38–41^. Multiple families of NIR dyes exist, and fine tuning of the physicochemical and biological properties can be achieved through structural manipulation of the dye scaffolds. Cyanine dyes are a versatile class of chromophore. The cyanine scaffold is comprised of two nitrogen containing heterocycles connected by a conjugated polymethine linker. This scaffold is amenable to modification; the heterocycles, the polyene linker, and the substituents can be manipulated to give rise to appealing optical properties and biological interactions. By manipulating discrete molecular characteristics and investigating the optoacoustic properties of the resulting dyes, effective contrast agents for optoacoustic imaging can be developed.

Nanoparticle delivery of contrast agents and therapeutics has gained increased appeal in recent years as novel nanostructures, are developed that display promising properties for clinical use. Mesoporous silica nanoparticles (MSN) have seen use in preclinical imaging studies due to their favorable biocompatibility and modifiable surface topology, which enables them to deliver molecules to tissues of interest^42^. Sequestration of drugs within the pores of silica nanoparticles has been utilized to achieve specific drug delivery of chemotherapeutics, which helps to avoid many deleterious off-target effects associated with those drugs^43^. In a recently published work, CaCO_3_-lentinan and silica nanoparticles were developed with a wormhole pore architecture^44,45^. It was found that the asymmetric nature of the pores gave rise to improved drug release kinetics in vivo. Specifically, the larger surface area allows for increased adsorption of cargo when compared to hexagonally ordered pores^46^, leading to highly controllable cargo retention and release compared to other nanoparticles. Furthermore, the use of silica nanoparticles allows for additional surface functionalization to improve tumor targeting beyond the basic enhanced permeability and retention (EPR) reported for nanoparticles, which only accounts for about 5% of the injected dose reaching the target and about 20–30% improved uptake versus normal organs^47–50^. With aforementioned benefits including biocompatibility, size, tunability, and high drug loading capacity, MSNs are quickly emerging as clinically viable nanomedicine for delivery of various agents. With that, the work described herein aims to develop an understanding of dye structure in optoacoustic imaging.

From our small library, a lead compound with strong optoacoustic signal has been identified and derivatized in order to produce more effective optoacoustic contrast agents. Throughout the process of preparing derivatives, the structural changes did not destroy the dyes’ optoacoustic performances. These dyes were encapsulated within tumor-targeted nanoparticles as potential contrast agents to detect pancreatic tumors in vivo using optoacoustic imaging in mice in real time and in 3D. In this study, we investigate the structural modifications of the dye to promote optimal encapsulation within the nanoparticle and to obtain detectable optoacoustic signal both in vitro and in vivo. Furthermore, our results support the definition of a chemical structure preferred for both in vitro and in vivo optoacoustic imaging studies in a pursuit to translate diagnostic agents to the clinic and address the need for early diagnosis and efficacious treatment of pancreatic cancers.

## Results and discussion

**Scheme 1:**
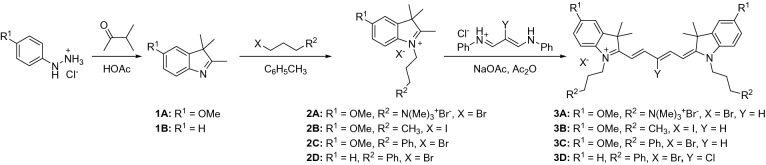
Synthesis of cyanine contrast agents **3A-D**.

As molecular characteristics that give rise to improved optoacoustic signal are poorly defined for small molecule dyes, the development of improved optoacoustic contrast agents was initiated first by screening a large library of pentametine cyanine dyes previously developed in our lab for fluorescence imaging applications (not all compounds shown). This library was prepared starting from commercially available phenylhydrazine derivatives, which undergo Fisher indole synthesis to create substituted indolenine derivatives **1** as outlined in Scheme 1. Alkylation of the indolenine heterocycles **1** with different alkyl halides such as propyl quaternary ammonium, butyl and phenyl propyl halides in toluene under reflux conditions gave the respective the heterocyclic salts **2A-2D**. Upon cooling, the reaction mixture to room temperature and subsequent purification via crystallization and co-precipitation with methanol and diethyl ether afforded the heterocyles **2** in good yields. The hygroscopic nature of heterocycles bearing quaternary ammonium moieties allowed us to perform alkylation of compound 2A under nitrogen atmosphere. In addition, precipitation of **2A** and **2B**, were a tedious, however, careful selection of solvents permitted a successful co-precipitation of these two compounds using methanol and acetone. As shown in Scheme 1, the final dyes **3A**–**3D** are pentamethine dyes and this require the use of the required pentamethine linker to efficiently synthesize them. Though the procedure to the synthesis of these scaffolds seems ubiquitous, development of the require conditions for their synthesis seems challenging, especially small molecules with net positive charges. To develop a facile and efficient approach in the synthesis of the final dyes, compound **3A** was obtained via condensation of the respective salt **2A** with malonaldehyde bisphenylamine salts in acetic anhydride under nitrogen condition to generate the final dye **3A**. UV–vis-NIR spectroscopy and TLC monitored the reaction progress. Upon consumption of the starting materials, the reaction mixture was allowed to cool to room temperature and diethyl ether was added to induce precipitation. The precipitate was collected by vacuum filtration. The crude solid was purified by recrystallization from methanol/acetone to yield the final dye **3A**. From that initial library, compound **3A** (Scheme 1), which contains the quaternary ammonium moieties of the nitrogen atom of the heterocyclic ring and methoxy groups at the 5 position of the benzene ring of the heterocyclic was identified as a promising lead due to its appealing optoacoustic properties.

**Figure 1:**
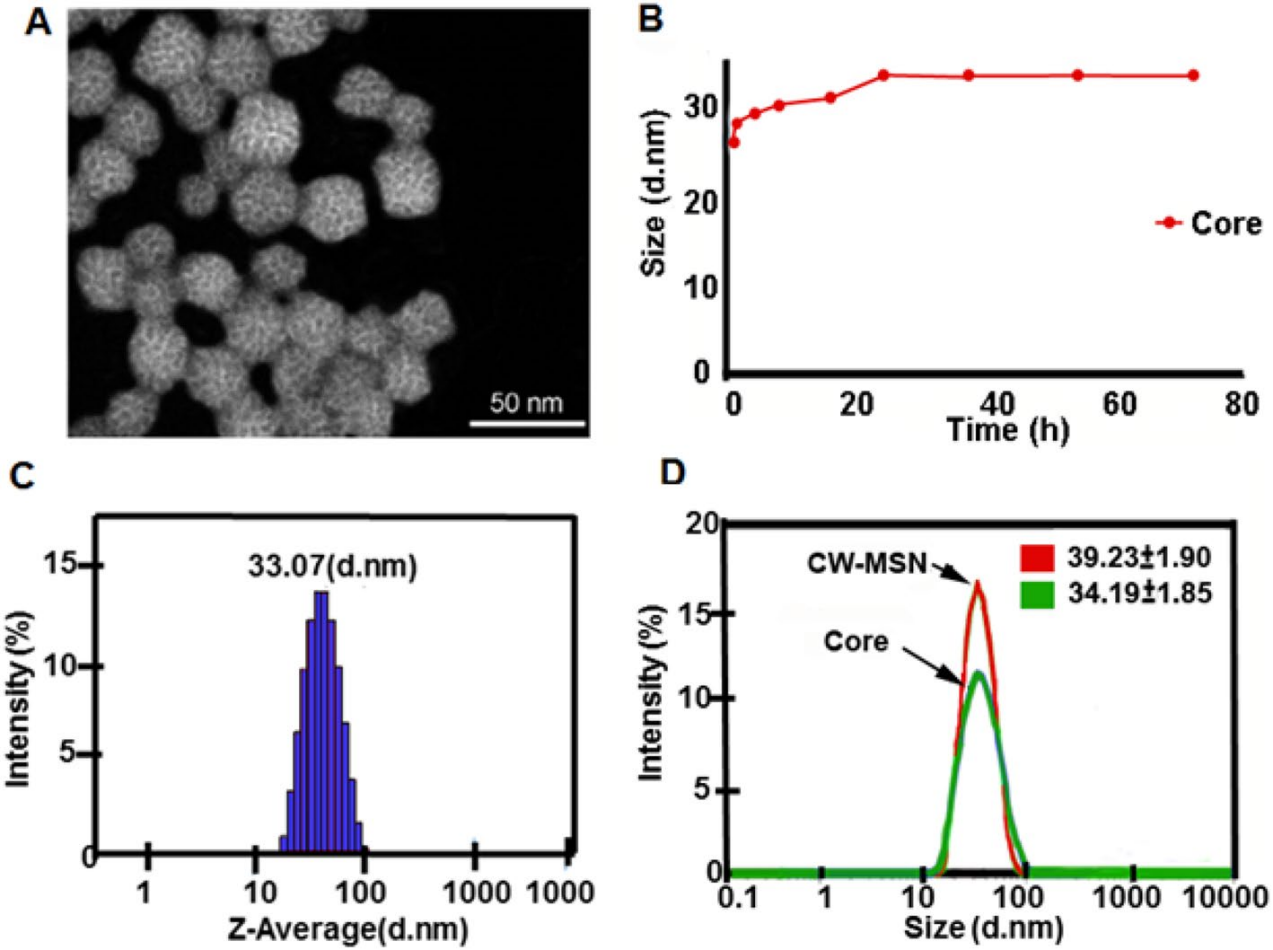
Characterization of the wormhole mesoporous silica nanoparticles (W-MSN). (**A**) Scanning transmission electron micrograph (STEM). Images of CW-MSN Core with an average particle size size of 26 ± 2.3 d.nm. Particles were measured using Image J https://imagej.net/Downloads. (**B**) Dynamic light scattering (DLS) results indicated that the MSN solution was monodisperse with a recorded PDI of 0.08 At 24 h, CW-MSN core reached their largest size (34.2 ± 2.0 d. nm), and further increases in size were not observed at 48 h and 72 h. (**C**) Dynamic light scattering indicates the average size of the particle at 24 h is 33.07 d nm. (**D**) Chitosan conjugation of the W-MSN (CW-MSN) increased the size of the W-MSN cores to 39.2 ± 1.9 d. nm.

The dyes were loaded into mesoporous silica nanoparticles (MSN). The asymetric wormhole mesoporous silica particles (W-MSN) were synthesized by a Sol–gel method and triethanolamine (TEA) was added to the mixture to result in asymetric wormhole pores^51–53^. The wormhole pores can encapsulate more cargo and release cargo at a slower rate than related hexagonal structures synthesized by a similar process^42^. Although a detailed mechanism of the growth formation of wormhole pores of W-MSN is unknown, it is hypothesized that complexing tetramethyl orthosilicate (TMOS) with TEA forms silatrane complexes under anhydrous conditions at elevated temperatures and limits the growth and aggregation of particles^52,54^. In contrast to many wormhole pore-based MSN particles, the present W-MSN have a smaller diameter (~ 33 nm) than the traditional > 50 nm nanoparticle; this should facilitate increased tumor uptake due to the smaller size. In order to achieve a smaller spherical mesoporous silica particle with wormhole pores, the procedure has key features including the use of TMOS instead tetraethyl orthosilicate (TEOS) and tetrapropyl orthosilicate (TPOS) as well as use of the polyalcohol base TEA as a substitute for NaOH or NH_4_OH (Fig. 1). This method creates a monodisperse (PDI < 1) population of MSNs. Following a series of acid and water dialysis, zeta potential of the solution is neutralized and particles are stable at room temperature for several months.

Initial experiments to encapsulate dye **3A** in silica nanoparticles revealed a weakness in the compound; while it gave a good optoacoustic signal, the high water solubility of **3A** led to leaching from the nanoparticles into the supernatant solution. Despite the appealing properties of the free dye, nanoparticles prepared from the lead compound lacked signal due to the poor retention of the dye. To overcome this issue, another derivatives **3B**–**3D** (Scheme 1) were prepared, which were increasingly hydrophobic. From the initial lead compound, quaternary ammonium groups were replaced with butyl groups to give dye **3B**. Similar replacement with a propyl phenyl groups gave compound **3C**. Lastly, removal of the heterocyclic methoxy groups of **3C** and the addition of a lipophilic *meso* chlorine atoms produced a new pentamethine cyanine compound **3D**.

**Figure 2:**
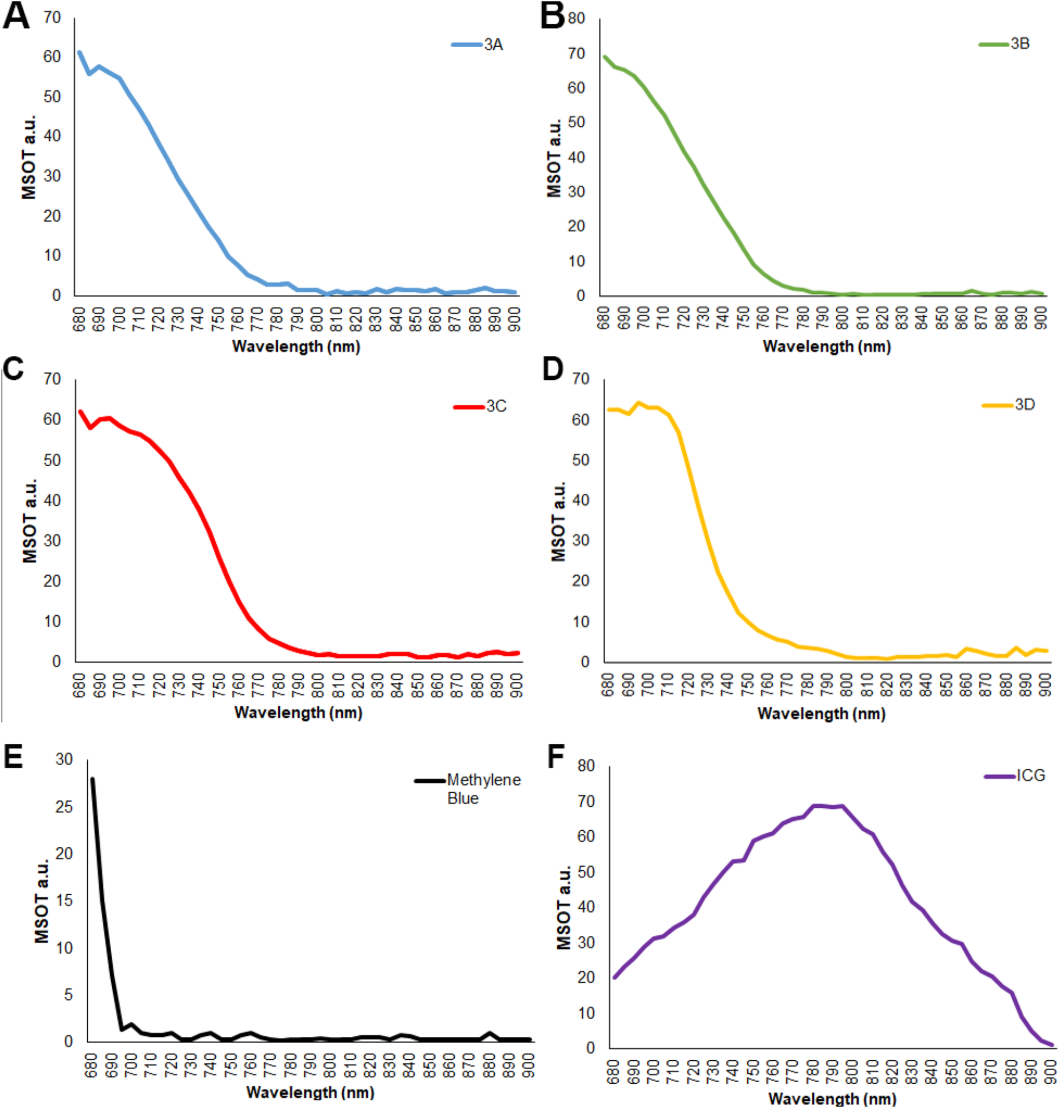
MSOT signal strength/dye concentration for the contrast agents **3A**-D and commercial dyes. At 1 mg/mL, each dye was evaluated in the tissue phantom using the MSOT system. The phantoms were scanned at 680 nm through 900 nm at 5 nm wavelength intervals. The graphs shown are raw MSOT data.

Compound **3D** showed an improved optoacoustic property for the in vivo imaging due its hydrophobicity as well as the addition of chlorine atom to establish the well-established concept of heavy atom effect. As shown in Fig. 2, the prepared compounds share similar optoacoustic spectral responses to initial lead **3A**. The MSOT spectra of **3A**-**D** fall between those of the two FDA approved NIR dyes, methylene blue (MB) and indocyanine green (ICG). Unlike MB, which sees a dramatic drop in signal after 700 nm, the signal from our new dyes extends much farther into the NIR region. Standard curves of **3A** and **3D** with concentration corresponding to loaded dye is found in Fig. 1S.

Retention of the dyes as measured by the change in absorbance of the supernatant after encapsulation improved as hydrophobicity increased (Table 1). Specifically, following dye encapsulation, the absorbance of the supernatant was measured. Higher signal in the supernatant was indicative of dye in solution and thus, less dye retention. With each modification, the retention of the dye in the nanoparticle improved, with only 9% of compound **3D** leaching out of the nanoparticles. Therefore, Compound **3D** was selected as our new lead compound, and compound **3A** used as the negative control in our study.

**Table 1:** Dye retention in silica nanoparticle vehicles.

Compound	Retention (%)
**3A**	25
**3B**	58
**3C**	65
**3D**	91

**Figure 3:**
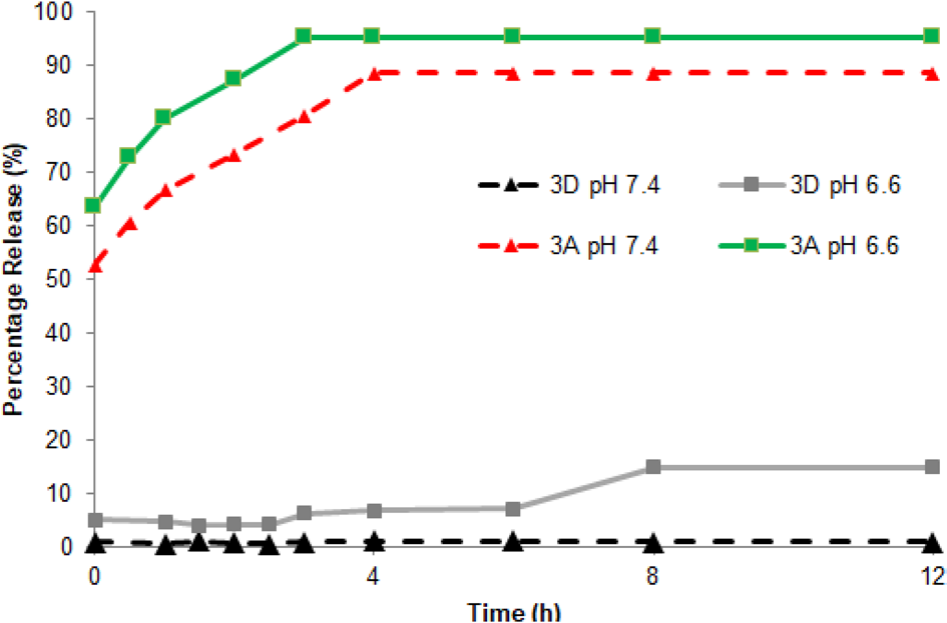
Compounds **3A** and **3D** release from nanoparticles at different pH values. Release is reported as a percentage (%) release compared to total fluorescence at the time of sample preparation prior to t = 0 h. Compound **3A** loaded nanoparticles show high initial dye release of 53% and 63% for pH 7.4 and 6.6, respectively, at t = 0 h. Release of compound **3A** increased (t = 0 h to t = 4 h) to and plateaued (t = 4 h to t = 12 h) at 89% and 95% for pH 7.4 and 6.6, respectively. Compound **3D** loaded nanoparticles released far less dye overall from t = 0 h to t = 12 h. Compound **3D** showed no release at pH 7.4, 0.9% at t = 0 h to 1.0% at t = 12 h, and a modest release of 5.2% to 6.9% at pH 6.6 t = 0 h to t = 4 h increasing to only 15% by t = 12 h.

Chitosan capping of the nanoparticles is designed to release the nanoparticle cargo in the mildly acidic extracellular matrix of the tumor. To explore the kinetics of pH dependent dye release, the nanoparticles loaded with dye **3A** or **3D** were washed with water until no absorption corresponding to the dye on UV–vis-NIR was detectable. The absorbance of the supernatant was monitored over time at pH values of 7.4 and 6.6 to mimic normal physiological pH and tumor pH respectively. In the release study shown in Fig. 3, compound **3A**, with permanently charged trimethylammonium groups on the pendant alkyl chains, showed excessive leaching of the dye from the nanoparticles at pH 6.6. Even at a normal physiological pH of 7.4, compound **3A** shows rapid release from the nanoparticles. Dye **3D**, the most hydrophobic of the compounds, showed limited release from the nanoparticles, even at a low pH.

**Figure 4:**
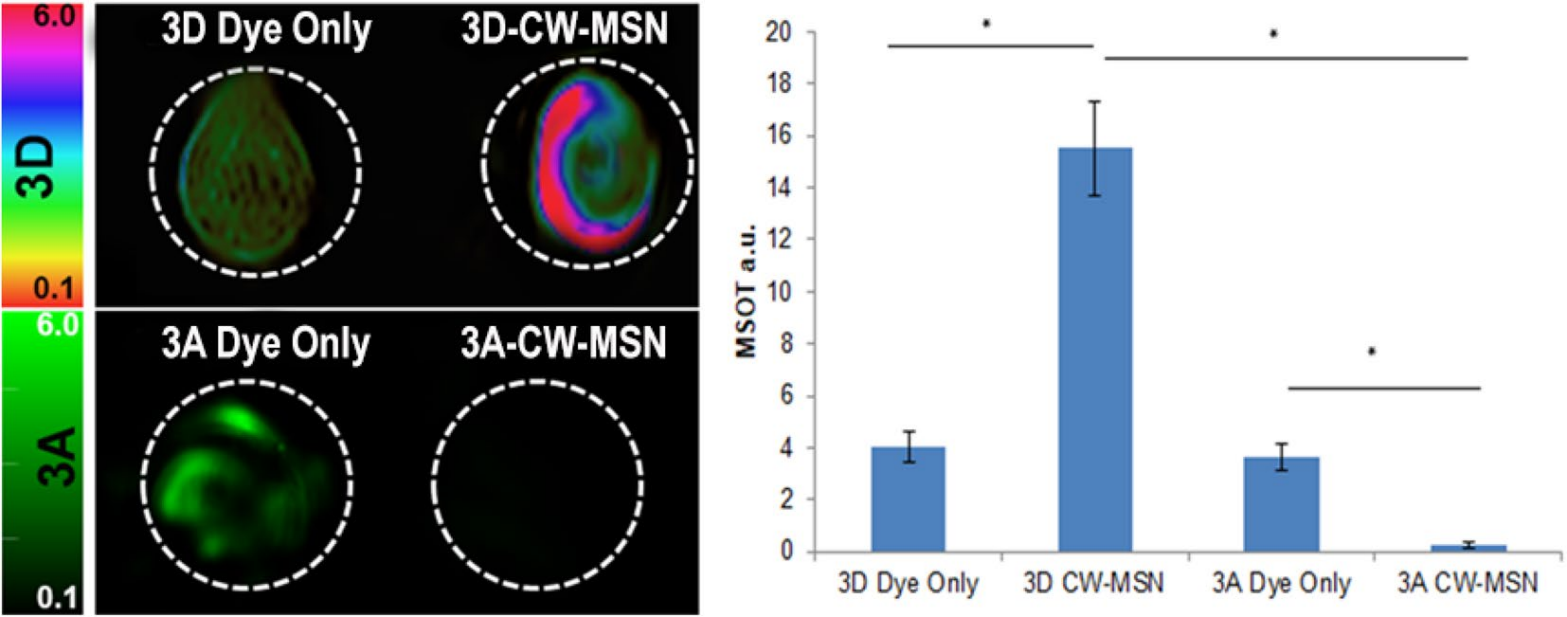
Optoacoustic imaging of tissue mimicking phantoms containing compounds **3D** (top) and **3A** (bottom) both as free dye (1 mM) (left) and as dye encapsulated in CW-MSN (0.1 OD) (right). Different colorbar scales are used to denote different dyes (HSB_HSL for compound **3D** and green for compound **3A**). Dyes **3A** and **3D** only controls showed similar MSOT signal strengths of 3.6 a.u. and 4.0 a.u., respectively. The optoacoustic signal presented by **3A** CW-MSN was significantly lower (0.23 a.u.) than that of **3A** dye only. An opposite trend was observed for comparison the optoacoustic signal of **3D** dye only control and the signal of **3D** CW-MSN, 15.5 a.u., which was significantly higher than both free **3D** dye (4.0 a.u. signal) and **3A** CW-MSN (0.23 X signal) *p* < 0.05. The retention of **3D** within the CW-MSN is likely due to the hydrophobic nature of the dye in comparison to the more hydrophilic **3A** dye.

**Figure 5:**
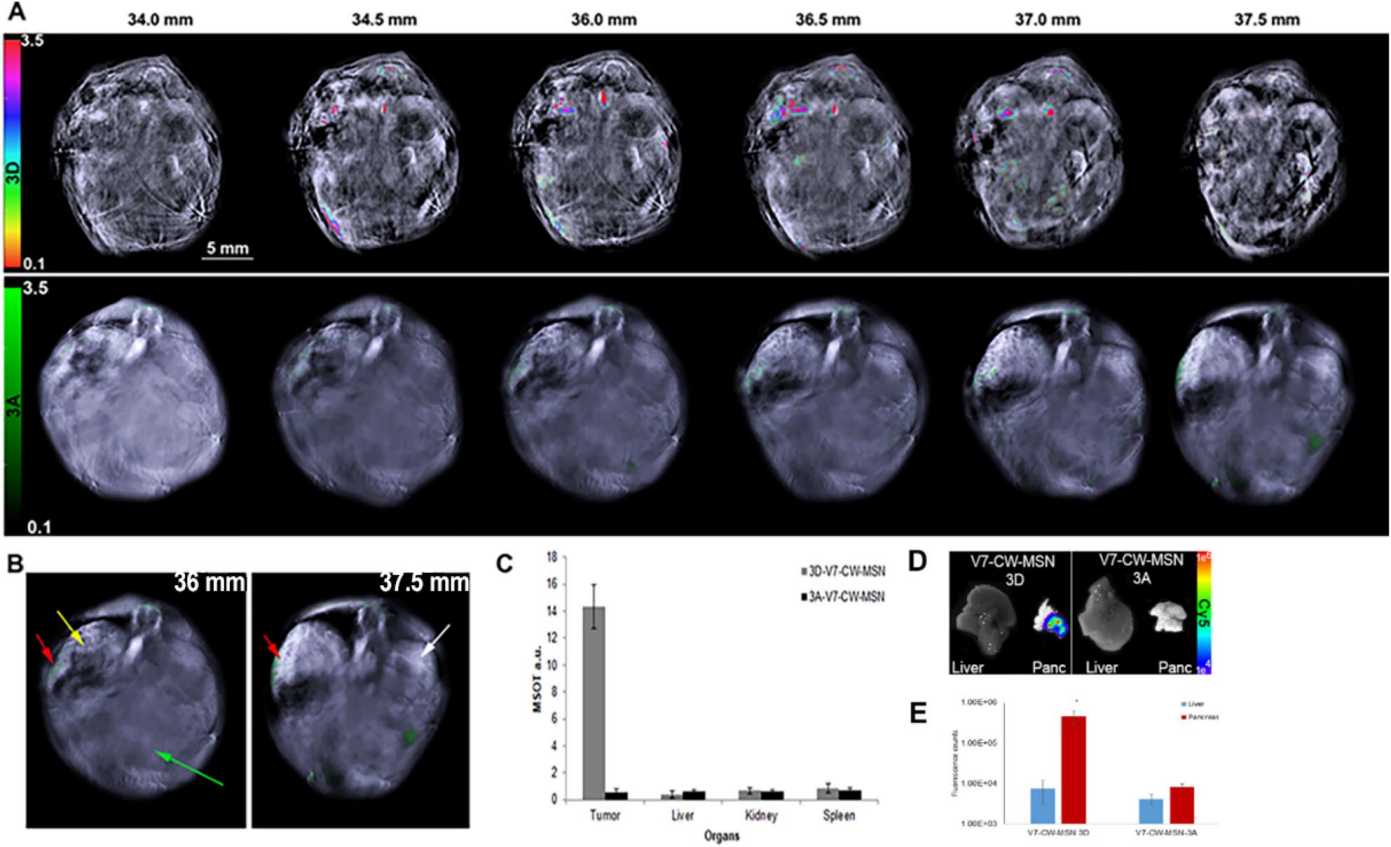
Serial slices (transverse) showing nanoparticle accumulation in orthotopic pancreatic tumor bearing athymic mice. Mice were IV tail-vein injected with 100 µL of **V7-CW-MSN** at a concentration of 5 μg/mL loaded with either **3A** or **3D**. The concentration of dye was balanced via an OD of 0.1 dye encapsulated within the **V7-CW-MSN**. 0.1 OD corresponds to a dye **3A** concentration of 150 μg/mL (132 million **3A** molecules per particle) and a **3D** concentration of 17.5 μg/mL (18 million 3D molecules per particle). Injected mice were imaged at an early time point of 4 h post-injection. Serial cross section of mice injected with **3A V7-CW-MSN** (bottom) or **3D V7-CW-MSN** (top) are presented together for comparison. Mean tumor size was 3 mm. (**A**) This image shows serial slices of mice injected with **3A V7-CW-MSN** (bottom) or **3D V7-CW-MSN** (top) as observed using MSOT. Differences in the background images can be attributed to mouse variation, where adjustments in the background image intensity need to be made in order to better visualize the blood vessels. In this case, the mouse imaged with **3A** is much girthier than the mouse imaged with **3D**. (**B**) Organs are identified by arrows: pancreas tumor (yellow), liver (green), spleen (red), kidney (white). (**C**) **3A V7-CW-MSN** optoacoustic signal was low overall and predominantly accumulated in the spleen (0.72 a.u.) with lower signal in the liver (0.61 a.u.), tumor (0.52 a.u.), and kidney (0.62 a.u.). **3D V7-CW-MSN** optoacoustic signal preferentially accumulated in the pancreatic tumor (14.3 a.u.), with lower signal corresponding to off-target accumulation for liver (0.42 a.u.), kidney (0.65 a.u.), and spleen (0.81 a.u.). Significant disparity in optoacoustic signal in the major blood vessels for **3A V7-CW-MSN** (0.98 a.u.) and **3D V7-CW-MSN** (12.1 a.u.) also appeared. (**D**) Ex vivo images of the liver and pancreas tumor confirmed signals using NIR fluorescence imaging. (**E**) Quantification of fluorescence signals from liver and pancreas. **V7 CW-MSN-3D** had significantly higher signal within the pancreas tumor (4.57E + 05 counts) compared to **V7 CW-MSN-3A** (8.36E + 03 counts) *p* > 0.0001.

To enable efficient trafficking of the nanoparticles to tumors, beyond the limited accumulation achieved by the EPR effect, the dye-loaded CW-MSN were surface functionalized with the V7 pH-low insertion peptide. In normal physiological conditions, the peptide does not interact with cells; however, tumor acidosis leads to protonation and folding of the peptide, forming a transmembrane alpha helix, which anchors it to the tumor cell membrane. Characterization of in vivo compound **3A** V7-CW-MSN and compound **3D** V7-CW-MSN, tumor accumulation was performed using MSOT imaging on 10 athymic mice bearing orthotopic pancreatic tumors of the cell line S2VP10. Note that S2VP10 cells contain a firefly luciferase and are not red shifted. Red-shifted S2VP10s would have an intrinsic fluorescent signal around 615 nm, adequate for photoacoustic microscopy but not for MSOT imaging^55,56^. Figure 5, presents a frontal view of transverse serial slices of the whole mouse body in 0.5 mm incremented step for mice injected with either compound **3A** V7-CW-MSN or compound **3D** V7-CW-MSN (juxtaposed). The cross-sectional view optoacoustic imaging of tissue mimicking intra-lipid-agar phantoms containing dye **3A**, and **3A** encapsulated in CW-MSN (**3A** CW-MSN), dye **3D**, and **3D** encapsulated in CW-MSN (**3D** CW-MSN) is presented in Fig. 4. A more complete comparison of **3A** and **3D** optoacoustic signal corresponding to OD is shown in Fig. 2S. The two free dye **3A** and **3D** controls show similar signal. The signal of **3A** CW-MSN is very weak compared to **3A** only. The dye leaching of **3A** observed during preparation of particles explains the absence of optoacoustic signal in **3A** CW-MSN in tissue phantoms. The optoacoustic signal of **3D** CW-MSN, however, was higher than its dye only analogue and considerably higher than that of **3A** CW-MSN. This result substantiates the proposed improved hydrophobicity for nanoparticle loading of **3D** over **3A**. The improved signal from **3D** CW-MSN over the free dye **3D** may originate from dye aggregation inside the nanoparticles. While aggregation-based quenching is undesirable for fluorescence applications, it has been shown to improve the optoacoustic performance of similar dye scaffolds^57^.

As shown in Fig. 5, higher mean signal per cross section was used as an indicator of tumor binding. Compound **3A** V7-CW-MSN showed low pancreatic tumor accumulation with slightly higher uptake in the liver and spleen. Concentration of optoacoustic signal was observed for **3A** V7-CW-MSN in the major blood vessels. Due to this low concentration, deoxy-hemoglobin was used for tumor segregation in compound **3A** V7-CW-MSN treated animals. Deoxy-hemoglobin MSOT images are shown in Fig. 3S. Given the early time point of imaging (4 h), this low concentration suggests that, in accordance with earlier results for both dye loading and tissue-mimicking phantoms, dye leaching of **3A** out of V7-CW-MSN occurred during sample preparation for mouse injection. **3D** dye again presents an oppositely situated case; MSOT imaging (4 h post-injection) of mice injected with **3D** V7-CW-MSN showed much higher major blood vessel optoacoustic signal than **3A** V7-CW-MSN as well as higher signal accumulation in the pancreatic tumor without the need for deoxy-hemoglobin segregation. Whereas off-target accumulation appeared higher, compared to pancreatic tumor signal accumulation for **3A** V7-CW-MSN, **3D** V7-CW-MSN showed higher signal accumulation in the pancreatic tumor than in the kidney, liver, spleen, and elsewhere. Inclusion of V7 was essential for tumor accumulation regardless of dye used, as shown in Fig. 4S.

## Conclusion

As optoacoustic imaging moves into clinical practice, there will be a need for contrast agents with strong signal and good biocompatibility. Organic dyes have revolutionized the potential of fluorescence imaging and are now poised to do the same for optoacoustic imaging; however, the underlying correlations between molecular structure and optoacoustic signal must be clearly defined. The series of pentamethine cyanine dyes discussed herein were prepared with the two-fold goal of having strong optoacoustic signal and sufficient retention in tumor-targeting nanoparticles for in vivo pancreatic tumor imaging. While all four compounds showed strong optoacoustic signal, the first of them, **3A**, proved too soluble in aqueous media and leached from the nanoparticles during preparation. With each successive modification to increase hydrophobicity, the retention improved. Hydrophobic dye with chlorine atom at the meso carbon **3D** showed the greatest retention in nano-vehicles for imaging and was retained even in acidic media. In both phantom and animal imaging studies, **3D** loaded nanoparticles gave superior signal, and, in in vivo imaging of orthotopic pancreatic tumors, clear foci were visible in the animals’ abdomens.

From this study, we do acknowledge the limitations of our tested probes **3A-3D** concerning their short wavelengths. For our future work, we will use our knowledge we have gained from developing the pentamethine library dyes (Cy5) and focus our efforts on designing the ideal heptamethine dyes (Cy7) for optoacoustic imaging.

## Experimental

### Chemicals and methods

All chemical reagents and solvents were purchased from Sigma Aldrich (Saint Louis, MO) and were used without further purification. Reaction progress was monitored on silica gel 60 F_254_ thin layer chromatography plates (Merck EMD Millipore, Darmstadt, Germany) NMR spectra were recorded on a Bruker Avance 400 MHz spectrophotometer in deuterated solvents purchased from Cambridge Isotope Labs (Andover, MA). All chemical shifts were recorded in parts per million (ppm) relative to the residual solvent. Signals are labelled as follows: s (singlet), d (doublet), t (triplet) m (multiplet) brs (broad signal) and coupling constants (*J*) are measured in Hertz (Hz). Mass spectra were performed at the Georgia State University Mass Spectrometry Facility using a Waters Q-TOF micro (ESI-Q-TOF) mass spectrometer ora Waters Micromass LCT TOF ES + Premier mass spectrometer. Melting points were recorded on a Mel-Temp apparatus and are not corrected. V7 pHLIP was synthesized by and purchased from CS Bio Company (Menlo Park, CA, USA). Dulbecco’s Modified Eagle’s Medium (DMEM), L-glutamine and Roswell Park Memorial Institute (RPMI 1640) medium were purchased from Life Technologies (New York, NY, USA) and dissolved in autoclaved PBS.

All methods were carried out in accordance with relevant guidelines and regulations of the *Scientific Report Journal*. The new compounds were synthesized and purified as outlined below. The purity of all synthesized compounds **3A-D** was confirmed with ^1^HNMR, ^13^CNMR and ESI-HRMS. These analytical methods used to determine the purity of compounds to be ≥ 95%.

### Synthesis of indolenine heterocycle

*5-methoxy-2,3,3-trimethyl indolenine*—**1a** was prepared as previously described and used without further purification^58^.

### Synthesis of heterocyclic salts

*5-methoxy-2,3,3-trimethyl-1-(3-(trimethylammonio)propyl)-3H-indolium bromide*—**2a** was prepared as previously described^59^.

*1-butyl-5-methoxy-2,3,3-trimethyl-3H-indol-1-ium iodide*—**2b** was prepared as previously described^60^.

*5-methoxy-2,3,3-trimethyl-1-(3-phenylpropyl)-3H-indol-1-ium bromide*—**2c** was prepared using the same procedure used to prepare **2d.**

*2,3,3-trimethyl-1-(3-phenylpropyl)-3H-indol-1-ium bromide*—**2d** was prepared as previously described^61^.

### Synthesis of polymethine linker

*N-((1E,2Z)-2-chloro-3-(phenylamino)allylidene) benzenaminium chloride*—**4** was prepared as previously described^62^.

### Synthesis of dyes 3A through 3D

The final dyes were prepared as previously described^59^. Briefly, indolium salts **2** (2 mol eq), sodium acetate (2 mol eq) and polymethine linker (malonaldehyde bisphenylamine hydrochloride its chloro derivative) (1 mol eq) were added to a dry, nitrogen flushed round bottom flask equipped with a stir bar. Acetic anhydride (5 ml) was added and the mixture was heated to 60 °C and stirred vigorously. Reaction progress was monitored by UV–vis spectroscopy and TLC. Upon consumption of the starting materials, the reaction flask was allowed to cool to room temperature and diethyl ether was added to induce precipitation. The precipitate was collected by vacuum filtration. The crude solid was purified by recrystallization from methanol/acetone or methanol/ether to give the pure final dyes. Following dye synthesis, 1 mM of each 3A-3D was added into a 1.3%w/w of agar and 6% v/v intralipid tissue-mimicking phantom to acquire spectral signatures with MSOT.

*5-methoxy-2-((1E,3E)-5-((E)-5-methoxy-3,3-dimethyl-1-(3-(trimethylammonio)propyl)indolin-2-ylidene)penta-1,3-dien-1-yl)-3,3-dimethyl-1-(3-(trimethylammonio)propyl)-3H-indol-1-ium bromide* (**3A**)—Blue solid; Yield 62%; m.p. 235–237 °C ; ^1^H NMR (400 MHz, MeOD) **δ** (ppm): 8.20 (t, *J* = 13.2 Hz, 2H), 7.42 (d, *J* = 8.8 Hz, 2 H), 7.12 (d, *J* = 2.4 Hz, 2H), 6.98 (m, 2H), 6.50 (d, *J* = 13.6 Hz, 2H), 4.25 (t, *J* = 7.6 Hz, 4H), 3.79 (m, 4H), 3.26 (s, 18H), 2.32 (br s, 4H), 1.74 (s, 12H); ^13^C NMR (100 MHz, MeOD) **δ** (ppm): 173.7, 159.9, 154.6, 144.3, 136.7, 127.5, 114.7, 112.7, 110.2, 104.6, 64.5, 56.5, 53.9, 28.1, 22.4; High-resolution ESI accurate mass spectra calculated m/3z for [C_39_H_59_N_4_O_2_]^3+^ 205.1541 found 205.1466. Also the m/z signal at 556.2771 is due to the loss of four -CH_3_ groups.

*1-butyl-2-((1E,3E)-5((E)-1-butyl-5-methoxy-3,3-dimethylindolin-2-ylidene)penta-1,3-dien-1-yl)-5-methoxy-3,3-dimethyl-3H-indol-1-ium iodide* **(3B)**—Blue solid; Yield 58%; m.p. 234 °C ; ^1^H NMR (400 MHz, CDCl_3_) **δ** (ppm): 8.04 (t, *J* = 13.1 Hz, 2H), 7.00 (m, 4H), 6.89–6.87 (m, 2H), 6.70 (t, *J* = 12.5 Hz, 1H), 6.18 (d, *J* = 13.64 Hz, 2H), 4.02 (t, *J* = 7.4 Hz, 4H), 3.86 (s, 6H), 1.77 (brs, 16H), 1.48 (q, *J* = 7.6 Hz, 4H), 1.00 (t, *J* = 7.3 Hz, 6H); ^13^C NMR (100 MHz, MeOD) **δ** (ppm): 171.6, 157.5, 152.6, 142.9, 135.6, 128.6, 113.6, 111.7, 109.1, 102.6, 55.9, 49.1, 43.3, 29.2, 27.2, 19.5, 13.8; High-resolution ESI accurate mass spectra calculated m/z for [C_45_H_51_N_2_O_2_]^+^ 527.3632 found 528.9456.

*5-methoxy-2-((1E,3E)-5-((E)-5-methoxy-3,3-dimethyl-1-(3-phenylpropyl)indolin-2-ylidene)penta-1,3-dien-1-yl)-3,3-dimethyl-1-(3-phenylpropyl)-3H-indol-1-ium bromide* **(3C)**—Blue solid; Yield 18%; m.p. 232 °C ; ^1^H NMR (400 MHz, CDCl_3_) **δ** (ppm): 8.20 (t, *J* = 12.8 Hz, 2H), 7.35–7.31 (m, 4H), 7.26 (brs [overlaps with solvent] 5H), 6.91 (s, 2H), 6.81–6.71 (m, 4H), 6.61 (t, *J* = 12.2 Hz, 1H), 6.18 (d, *J* = 13.5 Hz, 2H), 4.04 (brs, 4H), 3.83 (s, 6H), 2.83 (t, *J* = 7.2 Hz, 4H), 2.13 (brs, 4H), 1.76 (s, 12H); ^13^C NMR (100 MHz, MeOD) **δ** (ppm): 171.4, 157.4, 152.5, 142.7, 140.9, 135.4, 128.3, 128.1, 126.0, 124.5, 114.4, 113.4, 111.5, 109.0 102.4, 55.7, 55.4, 4.0, 42.9, 31.9, 28.6, 27.0; High-resolution ESI accurate mass spectra calculated m/z for [C_45_H_51_N_2_O_2_]^+^ 651.3945 found 651.1632.

*2-((1E,3Z)-3-chloro-5-((E)-3,3-dimethyl-1-(3-phenylpropyl)indolin-2-ylidene)penta-1,3-dien-1-yl)-3,3-dimethyl-1-(3-phenylpropyl)-3H-indol-1-ium bromide* (**3D**)—Blue solid; Yield 53%; m.p.125–127 °C; ^1^H NMR (400 MHz, DMSO-*d*_6_) **δ** (ppm): 8.41 (d, *J* = 13.6 Hz, 2H), 7.66 (d, *J* = 7.6 Hz, 2H), 7.45–7.41 (m, 4H), 7.34–7.22 (m, 12H), 6.20 (d, *J* = 13.2, 2H), 4.18 (t, *J* = 7.2 Hz, 4H), 2.79 (t, *J* = 7.2 Hz, 4H), 2.10–2.07 (m, 4H), 1.71 (s, 12H); ^13^C NMR (100 MHz, DMSO-*d*_*6*_) **δ** (ppm): 174.6, 147.9, 142.2, 141.9, 141.1, 128.9, 128.9, 128.6, 126.5, 126.0, 123.0, 122.8, 112.1, 100.2, 49.9, 44.0, 32.6, 28.6, 27.2; High-resolution ESI accurate mass spectra calculated m/z for [C_43_H_46_ClN_2_]^+^ 625.3344 found 625.3369.

### Synthesis of mesoporous silica nanoparticles

The mesoporous silica core of the nanoparticle began by mixing methanol (88 mmol/L, 100%), triethanolamine (TEA, 1.75 g/L, ≥ 99%), and Milli-Q-water (18.2 MΩ·cm at 25 °C). Cetrimonium bromide (CTAB) (8 g/L) at 80 ˚C (30 min) with constant stirring. The alkoxide precursor tetramethyl orthosilicate (TMOS 11 mmol/L, ≥ 99.0%) was added dropwise to the solution followed by stirring vigorously for 24 h at 80 °C. The excess of the CTAB was removed from the mesoporous silica core using dialysis with a 1:1 ratio of methanol and Milli-Q- water. This resulted in the wormhole pored mesoporous silica (W-MSN) core component of the nanoparticle.

### Chitosan capping of the W-MSN Core to result in CW-MSN

A gatekeeping component is required for mesoporous particles to facilitate encapsulation and release of the dye within the mesoporous silica particle. Chitosan (1%) was dissolved in 5% acetic acid (200 mL) using constant stirring for 24 h at 25 °C. The chitosan gatekeeping component was added to the surface of the W-MSN core particles by initially dispersing the W-MSN in ethanol and separating them using ultrasonication (3 min). Next, the solution pH was decreased from pH 3.8 to 3.5 with acetic acid and the 0.1 g of 3-glycidoxypropylmethyldie-thoxysilane (GPTMS) was added to the solution, which was stirred for 3 h to result in a reactive surface of the W-MSN core. The chitosan solution (1%) was added to the reactive W-MSN core at 25 °C with constant stirring for 24 h resulting in CW-MSN. The CW-MSN particles were washed with ethanol (70%) and stored at 4 °C.

### CW-MSN conjugation with the V7 peptide (V7-CW-MSN)

The CW-MSNs were functionalized by adding 400 μM SMCC (succinimidyl 4-(N-maleimidomethyl) cyclohexane-1-carboxylate) (Sigma-Aldrich, St. Louis, MO, USA) to 10 mg/2 mL and stirred for 10 min. Following functionalization, V7 pHLIP peptide (456 μM) was added to the functionalized CW-MSN with constant stirring for 16 h. The V7 pHLIP was synthesized by CS Bio with a sequence of ACEEQNPWARY LEWLFPTETLLLEL (Menlo Park, CA, USA). Following V7 conjugation, the solution is used immediately as stability complications are likely outside 24–48 h.

### Characterization of the CW-MSN Core

The CW-MSN Core was characterized using Dynamic Light Scattering and transmission electron microscopy. The Dynamic Light Scattering (DLS) was conducted using a Zetasizer Nano ZS (Malvern Instruments, UK) with a 5 mW HeNe laser at 633 nm wavelength. The synthesized TMOS-based particles were suspended in Milli-Q water (1.5 mL/L of synthesis). The ultrapure water preserved at room temperature was used in the data analysis with a refractive index of 1.33 and viscosity 0.89 in automatic mode using the 6.0 Zetasizer Nano software. The morphological characterization of the W-MSN was performed with transmission electron microscopy (TEM). Morphological characteristics such as average size and shape were qualitatively analyzed with a Zeiss Supra 35VP electron microscope (Zeiss, Pleasanton, CA, USA). W-MSN were analyzed by placing 10 μL of the sample on a SEM studs and, before viewing, wick dried using filter paper. The average pore size of the W-MSN were calculated based on analysis of 26 CW-MSN Core particles using the Image J software (https://imagej.net/Downloads).

### Media preparation for the in vitro nanoparticle assays

To evaluate the pH specificity of the nanoparticles in vitro, a phosphate buffered, pH specific media was created that was independent of bicarbonate buffering. A 25 mM phosphate buffer was prepared at pHs 7.4 or 6.6 by dissolving sodium phosphate monobasic and dibasic solution dissolved in distilled water (Sigma-Aldrich, St. Louis, MO, USA). The phosphate buffer was sterilized using an autoclave. This phosphate buffer was the base of both the pH specific media and the washing solution. The pH specific media contained the 25 mM phosphate buffer, 13.6 g of DMEM powder, 10% fetal bovine serum, and 1% L-glutamine and filtered through grade-1 Whatman qualitative filter paper (Sigma-Aldrich, St. Louis, MO, USA). The solution pH was confirmed using a pH meter (Denver Instrument Ultrabasic, Bohemia, NY, USA). The washing solution also contained the same 25 mM pH specific phosphate buffer and included 1% fetal bovine serum. The pH of the solutions was adjusted with sterilized sodium hydroxide (1 M) or hydrochloric acid (1 M) as needed.

### In vitro assay to determine the ability of CW-MSN for entrapping either 3D or 3A as cargo for optoacoustic detection

Either 0.1 mM of dye or 0.1 OD of dyes (150 μg/mL 3D or 17.5 μg/mL 3A) within the CW-MSN nanoparticles were added to the agar gel-based tissue mimicking phantoms as in previous studies^25,60^. Tissue mimicking phantoms were prepared from 1.3%w/w of agar and 6% v/v intralipid added to DI water which results in creating a gel with a reduced light scattering coefficient of μ = 10 cm^−1^. **3D** dye only, **3D** CW-MSN, **3A** dye only, and **3A** CW-MSN were added to the 3 mm diameter cylindrical opening in the tissue phantoms. All samples were evaluated at multiple wavelengths (680, 710, 730, 740, 760, 760, 770, 780, 800, 850 and 900 nm). At least 20 average readings were taken during the analysis at each wavelength. Quantitative measurements were conducted using the Region of Interest method using ViewMSOT software version 3.8 **p < 0.01.

### Orthotopic implantation of Pancreatic Cancer Cells within athymic mice

Four-week old female athymic mice 4 weeks of age were purchased from Envigo (Envigo Laboratories, Indianapolis, IN, USA) and study approved by Wake Forest University Institutional Animal Care and Use Committee (IACUC). To reduce potential imaging background, mice were fed 2920X alfalfa free-rodent diet (Envigo Laboratories, Indianapolis, IN, USA). Mice were orthotopically implanted with S2VP10L pancreatic cancer cells as previously described^63^. S2VP10L cells contain traditional firefly luciferase, and are not red shifted. For the surgical procedure, mice were anesthetized with isoflurane at 1.5% in 1 L O_2_ and betadine was used to disinfect the skin over the abdomen. An incision into the left upper quadrant allowed for identification of the spleen and pancreas. A solution of 5 × 10^5^ S2VP10L cells/30 μL of serum-free DMEM media was injected into the pancreas using a 28G syringe. To prevent leakage of the tumor cells, a sterile cotton tipped applicator was held over the injection site for 30 s. The pancreas and spleen were returned to the abdomen and the wound was closed in a single layer using 5–0 nylon sutures. Mice recovered underneath a warming blanket and were returned to their cages.

### Tumor monitoring with bioluminescence imaging

To evaluate potential leakage of cells from orthotopic implantation, bioluminescence imaging was performed immediately following surgery with the advanced molecular imager instrument (AMI) (Spectral Imaging Instruments, Tucson, AZ, USA). Mice were imaged 10 min after IP injection of 2.5 mg luciferin. Any showing signs of peritoneal leakage were excluded from further study. After 5 days, the sutures were removed to prevent artefacts in future imaging studies. Tumor size was followed daily with bioluminescence imaging. At day 7 post-implant, mice bearing S2VP10L were sorted based upon tumor signal so that each group had equal group mean signal based upon bioluminescence signal to evaluate **3D** V7-CW-MSN and **3A** V7-CW-MSN for in vivo imaging using MSOT.

### Evaluation of 3D and 3A V7-CW-MSN accumulation in vivo assessed using multispectral optoacoustic tomography

Detection of **3D** V7-CW-MSN and **3A** V7-CW-MSN biodistribution within orthotopic pancreatic xenografts was performed using the inVision-512 echo multispectral optoacoustic tomography system containing ViewMSOT version 3.8 software (iThera Medical, Munich, Germany)^45^. Anesthesia for the mice consisted of 1.5% isoflurane inhalant delivered in 0.8 L of medical air and 0.1 L of O_2_. Anesthetic depth was maintained throughout the image acquisitions. Mice were imaged with the ventral side up in the MSOT animal holder. All mice were imaged using MSOT prior to IV injection of **3D** V7-CW-MSN and **3A** V7-CW-MSN to determine baseline signal. MSOT imaging shows optimal detection at earlier time points^25,25,50^. MSOT imaging was performed on mice 3 h post IV injection of **3D** V7-CW-MSN and **3A** V7-CW-MSN, using transverse slices with a 0.2 mm step from the liver to the kidney (32–47 mm), at wavelengths of 680, 710, 730, 740, 760, 770, 780, 800, 850, 900 nm. Twenty-five averages per wavelength were utilized to minimize the influence of breathing and animal movement. The acquisition time was 10 μs per frame. Pancreatic tumors were identified based upon the deoxy-hemoglobin signal in the live-screen multispectral preview in ViewMSOT 3.8^45^.

### MSOT image reconstruction and analysis

Raw data obtained with MSOT were reconstructed with multispectral analysis performed as previously described^20,63^. Spectral analysis was performed at wavelengths corresponding to **3A** or **3D** dye within the nanoparticles and oxy- and deoxy-hemoglobin. Spectra utilized for spectral unmixing of the nanoparticle are located within Fig. 2 with the spectral shape of **3A** in blue and **3D** in yellow. Both oxy- and deoxy-hemoglobin spectra were utilized as identified in previous publications^64,65^. Version 3.8 of ViewMSOT software (iThera Medical, Munich, Germany) was used to reconstruct MSOT images from the raw data using a backprojection algorithm at a resolution of 75 μm. Backprojection reconstruction is a very fast, semi-quantitative approach to display the anatomical features of the image, an in-depth description of the algorithm can be found here^66^. The multispectral processing was conducted using linear regression with ViewMSOT 3.8, where known molar absorptivity spectra (e.g., for **3D** dye, **3A** dye, oxy-, and deoxy-hemoglobin) are used to model the relationship between chromophore concentration and MSOT signal over a range of wavelengths. The approach assumes knowledge about all absorbers present in the imaged tissue in order to correctly attribute contributions from the different wavelengths to the unmixed component images. In order to ensure comparability among data sets, the reconstruction parameters (field of view, speed of sound, pixel size, and the high/low pass filters) and spectral unmixing parameters were consistently applied to all data. Spectral unmixing was performed in the absence of correction for fluence heterogeneities and attenuation as a function of tissue depth including spectral coloring.

The orthotopic tumors had a similar location and distance from the skin surface from animal to animal, thus, fluence issues would equally affect all animals. The location of orthotopic tumors was identified based upon the presence of deoxy-hemoglobin, upon deoxy-hemoglobin spectra, and upon relative location of the spleen and kidney. In addition, a region of interest (ROI) method was applied to determine signal strength in the tumors, liver, and kidney of both **3D** V7-CW-MSN and **3A** V7-CW-MSN treated mice using ViewMSOT software and reported as MSOT a.u. The ROI was manually created with an ellipse drawing tool with location of tumor based upon deoxy-hemoglobin component. The deoxy-hemoglobin component was used to provide a basis for tumor segmentation in animals receiving **3A** V7-CW-MSN as very little **3A** signal was detected (Fig. 5). The ROI area was kept constant at 3.5 mm^2^ for all image slices, thus creating a nonuniform elliptical prism volume of interest (VOI). The mean pixel intensity per cross-section in the VOI for the spectrally unmixed injected agent (compounds **3D** and **3A** control) was plotted as MSOT signal vs position to assess the signal strength in the tumor. This analysis produced a consistent parabolic shape of signal over distance in the tumor. The maximal “mean signal per cross-section” in the volume was used as a quantitative indicator of probe binding in the tumor. Because optoacoustic signals using the detection geometry of this system are subject to out-of-plane contributions, this method was used to find the center of signal intensity and minimize variability from out-of-plane artefacts. The capacity of this optoacoustic system to deliver semiquantitative data reflective of relative probe accumulation in vivo in murine models using the aforementioned reconstruction and multispectral unmixing methods was previously established^20,25,49^. The MSOT a.u. values for **3D** V7-CW-MSN and **3A** V7-CW-MSN control were compared using SAS 9.3 (SAS, Cary, NC, USA).

### Statistics

In vitro, comparison of signal intensity of **3D** dye and **3A** dye alone and within the nanoparticle was performed with T-test using SAS 9.3 (SAS, Cary, NC, USA). In vivo, the MSOT a.u. values for **3D** V7-CW-MSN and **3A** V7-CW-MSN control were compared using Wilcoxon sum-rank test and ANOVA followed by Tukey post hoc test using SAS 9.3 (SAS, Cary, NC, USA). Significance was observed where *p* < 0.05.

## Supplementary Information

Supplementary Information.
