# Supplementary material for: Improved pentamethine cyanine nanosensors for optoacoustic imaging of pancreatic cancer
Source: Sci Rep. 2021 Feb 23;11:4366. doi: 10.1038/s41598-021-83658-3 (PMC7902650; doi:10.1038/s41598-021-83658-3)
Supplement: Supplementary file 1 — Supplementary Information. [file 41598_2021_83658_MOESM1_ESM.docx]

Supporting Information

**Improved Pentamethine Cyanine Nanosensors for Optoacoustic Imaging of Pancreatic Cancer**

Matthew D. Laramie,^[1†]^ Benjamin L. Fouts,^[2,3†]^ William M. MacCuaig,^[3,4]^ Emmanuel Buabeng^[1,5]^, Meredith A. Jones,^[4]^  Priyabrata Mukherjee,^[6]^ Bahareh Behkam,^[7]^ Lacey R.McNally,^[2#,3,4,8,*]^ Maged Henary^[1,5,#,*]^

^1^ Department of Chemistry, Georgia State University, Atlanta, GA 30303, USA.

^2^ Department of Surgery, Oklahoma Health Science Center, Oklahoma City, 73104, USA.

^3^ Stephenson Cancer Center, Oklahoma Health Science Center, Oklahoma City, OK 73104, USA.

^4^ Department of Biomedical Engineering, University of Oklahoma, Norman, OK 72073, USA.

^5^ Center for Diagnostics and Therapeutics, Georgia State University, Atlanta GA 30303, USA.

^6^ Department of Pathology, Oklahoma Health Science Center, Oklahoma City, OK 73104, USA.

^7^ Department of Mechanical Engineering, Virginia Tech University, Blacksburg, VA 24061, USA.

^8^ Department of Cancer Biology, Wake Forest University, Winston-Salem, NC 27157, USA.

† Co-first author.

# Co-senior author.

* To whom correspondence should be addressed (MH: mhenary1@gsu.edu; LM: lacey_mcnally@hotmail.com)

**Table of Contents**

S1 – ^1^H NMR Spectrum of compound **3A**

S2 – ^13^C NMR Spectrum of compound **3A**

S3 – Mass spectrum of compound **3A**

S4 – ^1^H NMR Spectrum of compound **3B**

S5 – ^13^C NMR Spectrum of compound **3B**

S6 – Mass spectrum of compound **3B**

S7 – ^1^H NMR Spectrum of compound **3C**

S8 – ^13^C NMR Spectrum of compound **3C**

S9 – Mass spectrum of compound **3C**

S10 – ^1^H NMR Spectrum of compound **3D**

S11 – ^13^C NMR Spectrum of compound **3D**

S12 – Mass spectrum of compound **3D**

Figure 1S – A) Dye **3A** standard curve of absorbance (OD) vs. concentration (ug/mL). B) Dye **3D** curve of absorbance (OD) vs concentration (ug/mL). C) Amount of dye encapsulated within MSNs for OD measures of 0.1 in terms of concentration and dye molecules per MSN particle.

Figure 2S – Varying the concentrations of **3A** and **3D** using MOST

Figure 3S – Showing deoxy-hemoglobin of mice for Figure 5A

Figure 4S – Showing MOST images in mice injected with silica nanoparticle containing either **3A** or **3D** with no V7 targeting (control).


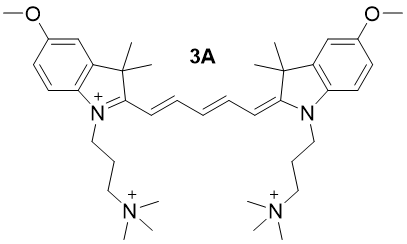


S1. ^1^H NMR Spectrum of compound **3A**


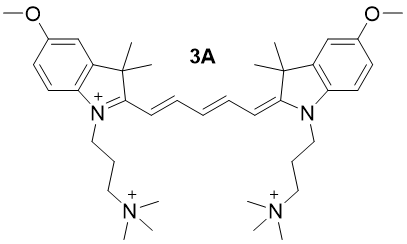


S2. ^13^C NMR Spectrum of compound **3A**

M/3Z


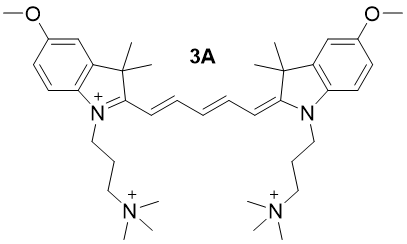


Molecular Weight: 615.93

M/2Z

**S3.** Mass spectrum of compound **3A**


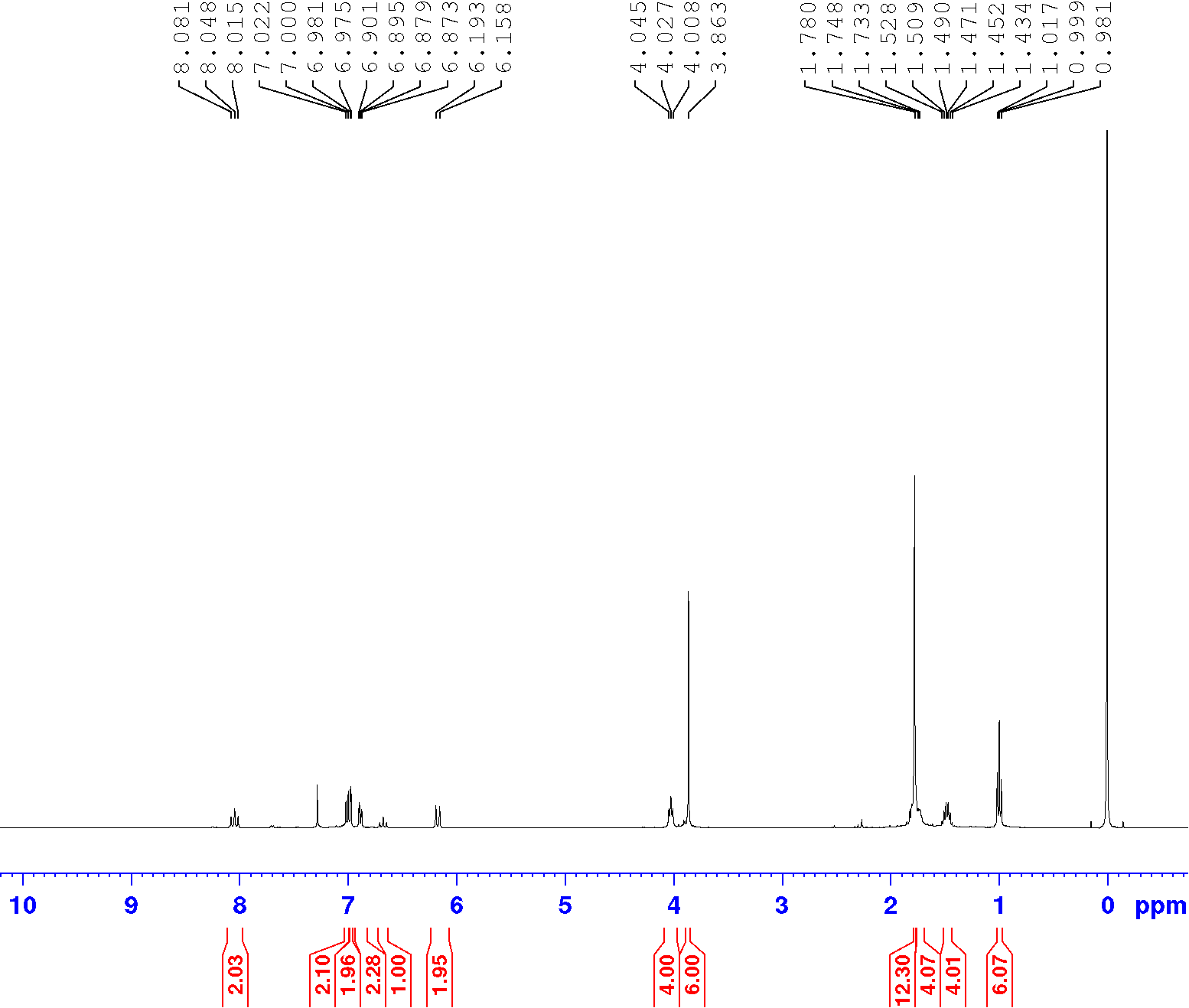

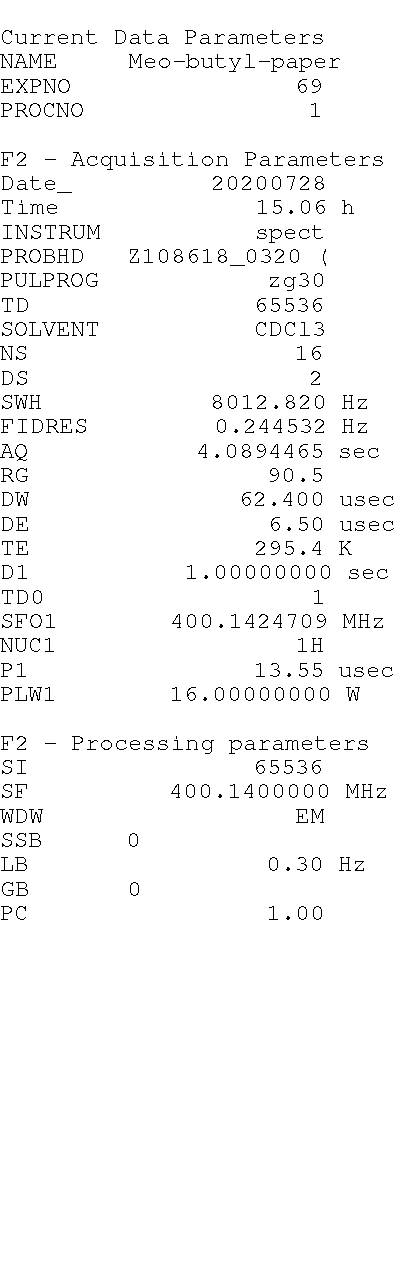


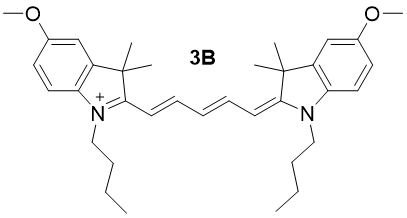


S4. ^1^H NMR Spectrum of compound **3B**


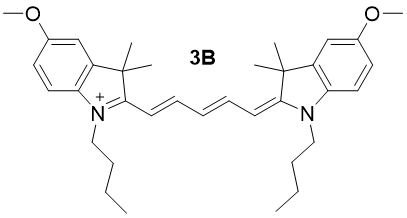

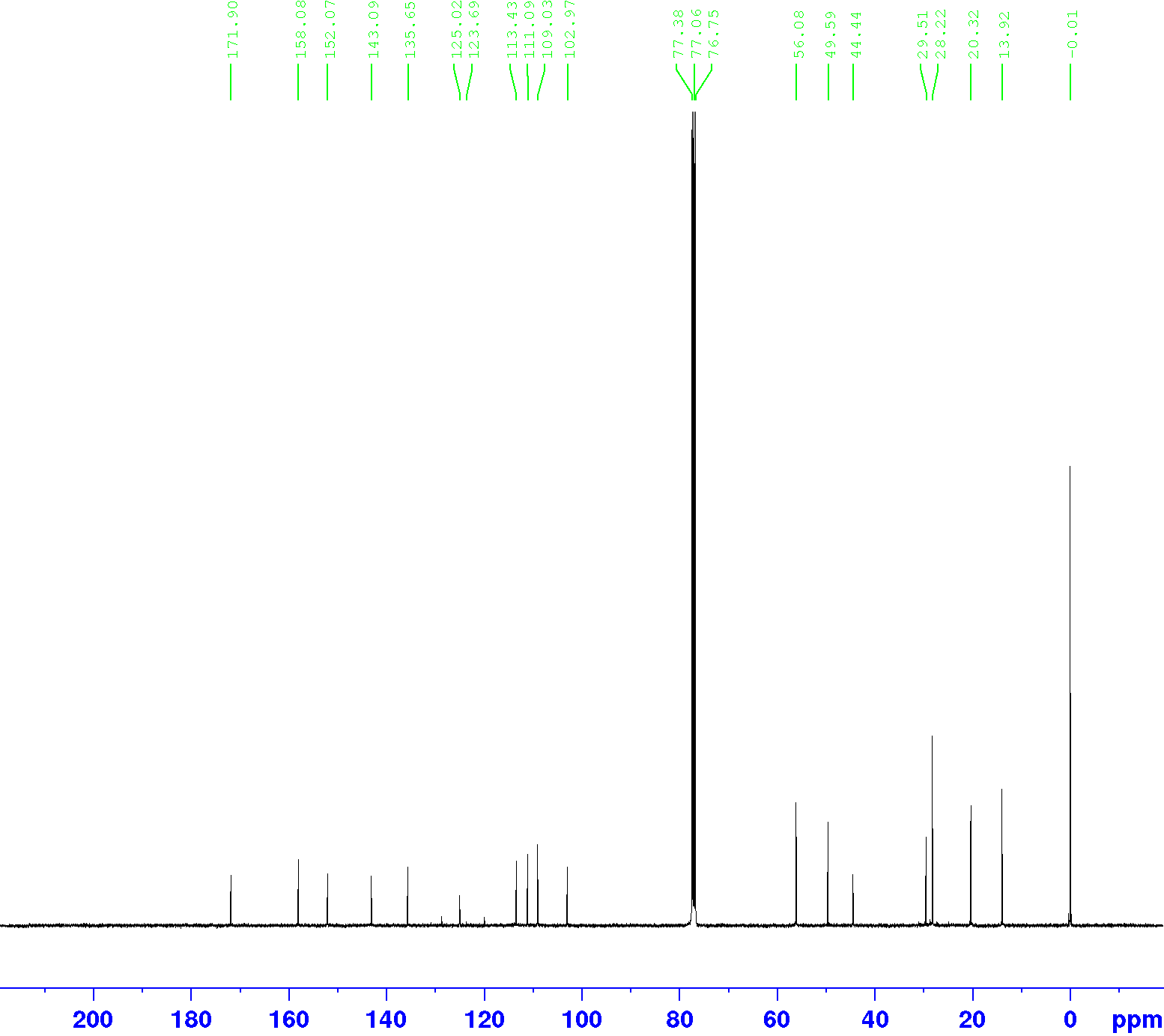


S5. ^13^C NMR Spectrum of compound **3B**


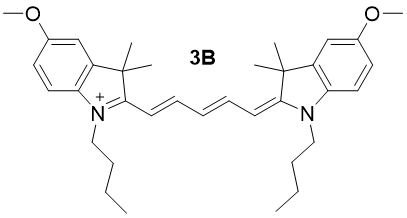
S6. Mass spectrum of compound **3B**

Molecular Weight: 527.77

**
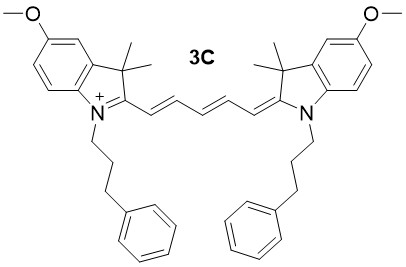
**
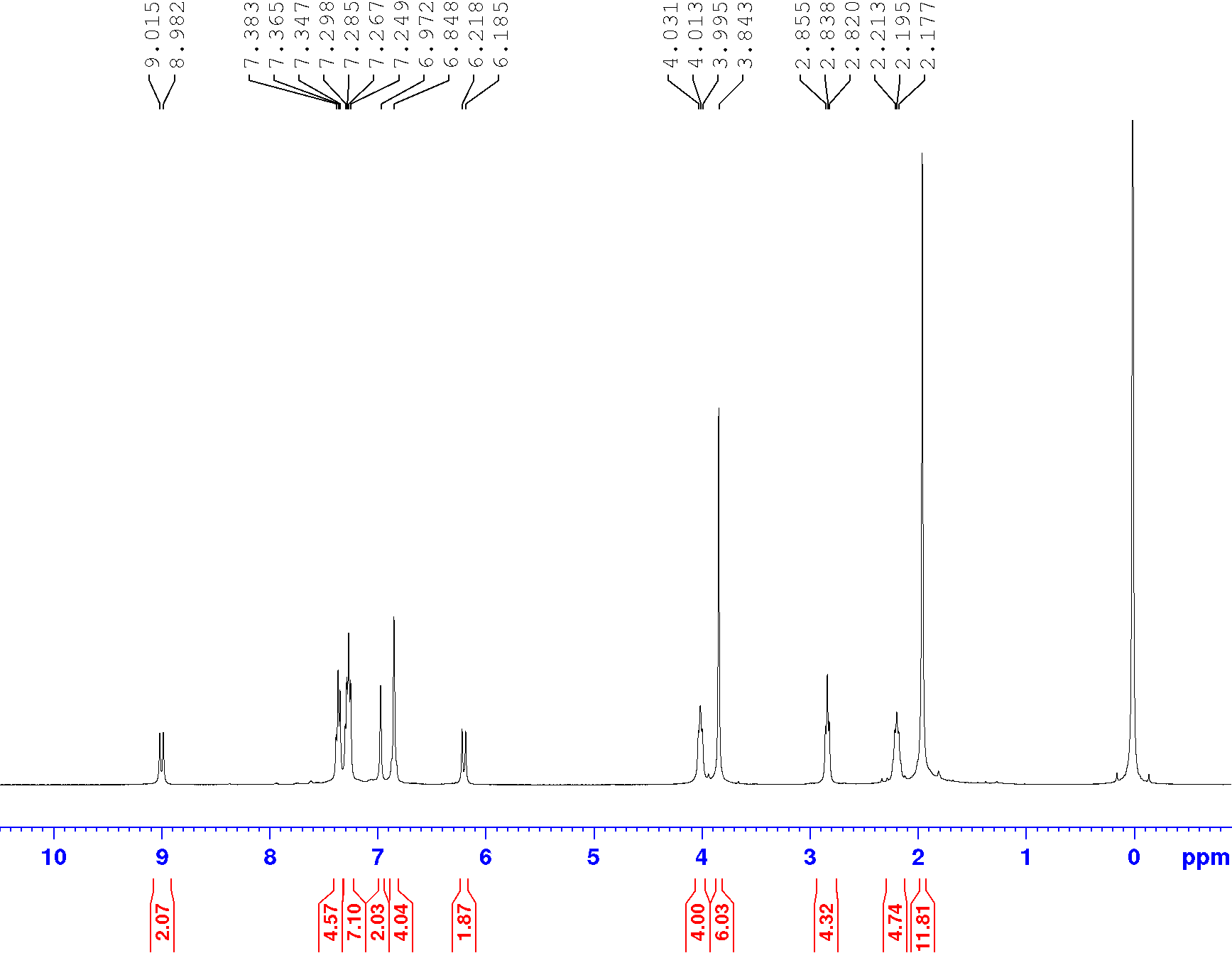

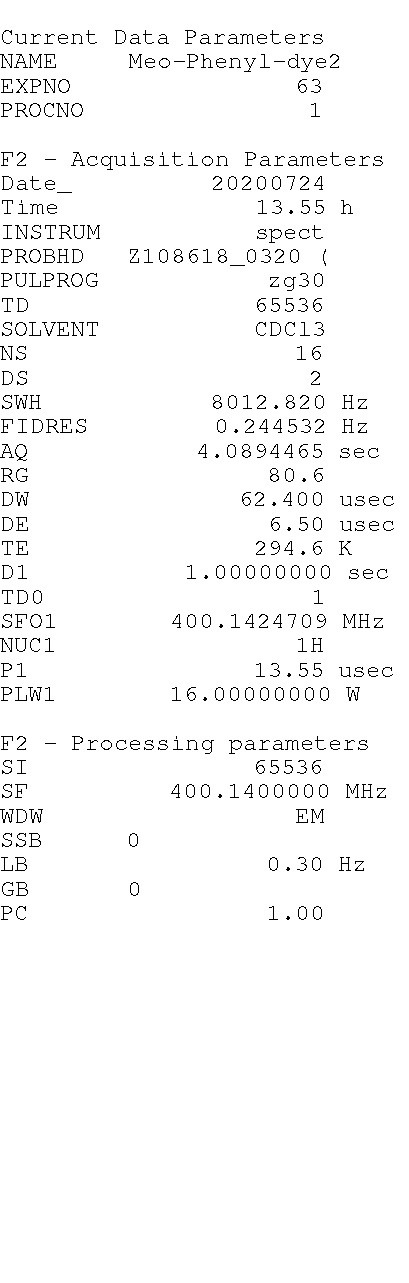


S7. ^1^H NMR Spectrum of compound **3C**

**
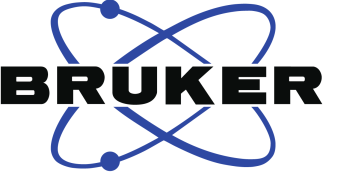
**

**
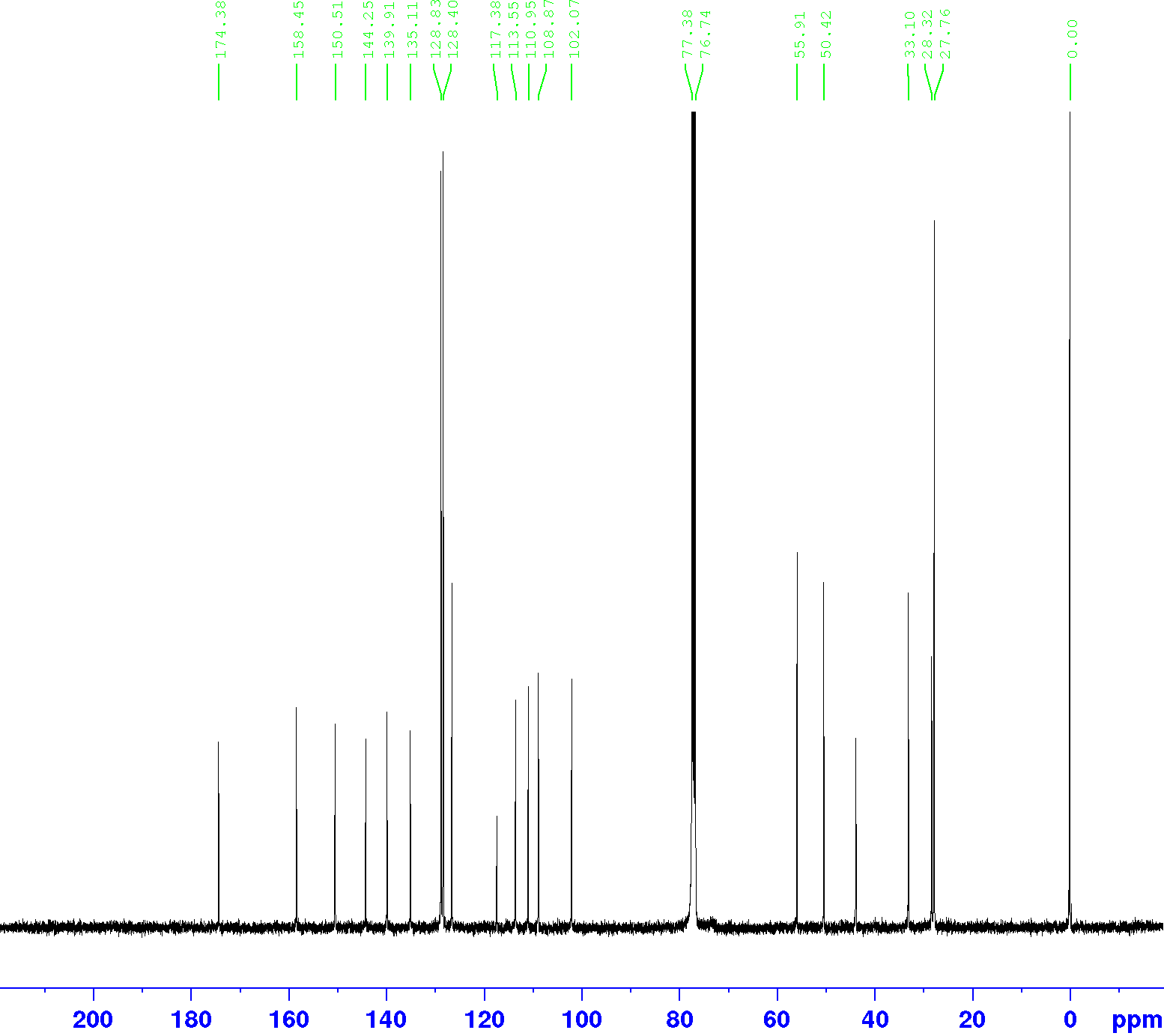
** **
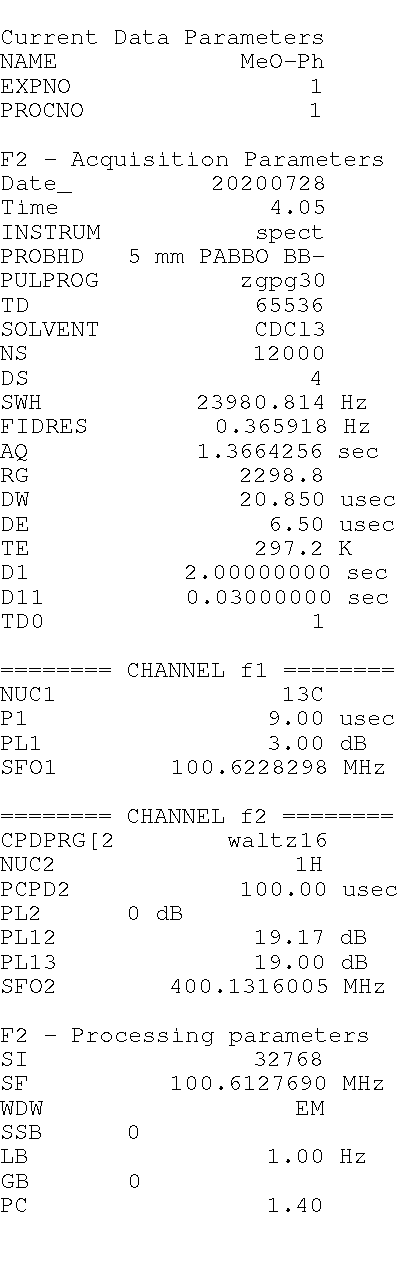
**


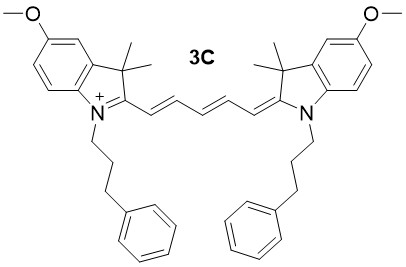


S8. ^13^C NMR spectrum of **3C**


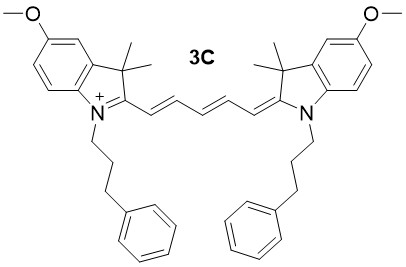


Molecular Weight: 651.91

**S9.** Mass spectrum of compound **3C** Molecular Weight: 651.91

**
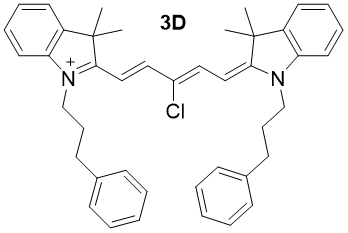
**S10. ^1^H NMR Spectrum of compound **3D**

**
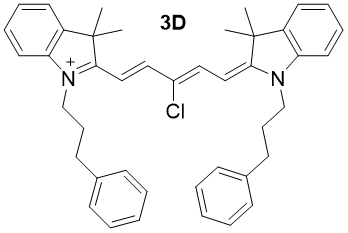
**S11. ^13^C NMR Spectrum of compound **3D**


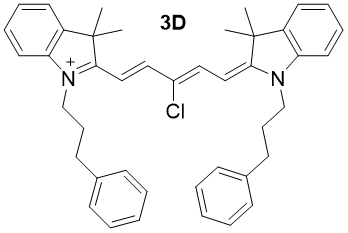
S12. Mass spectrum of compound **3D**

Molecular Weight: 626.30


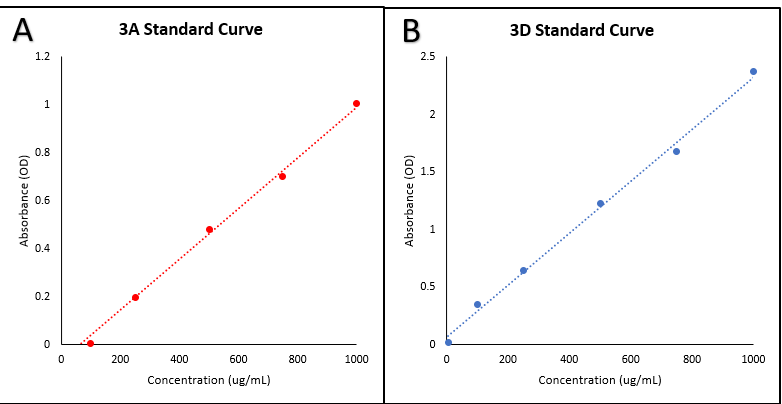

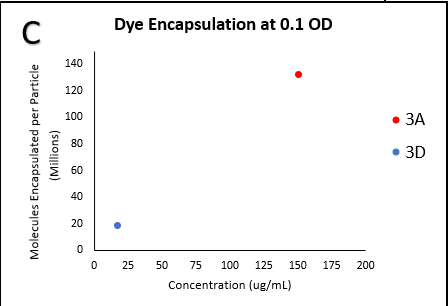


Figure 1S. A) Dye **3A** standard curve of absorbance (OD) vs. concentration (ug/mL). B) Dye **3D** curve of absorbance (OD) vs concentration (ug/mL). C) Amount of dye **3A** or **3D** encapsulated within MSNs for OD measures of 0.1 in terms of concentration and dye molecules per MSN particle.


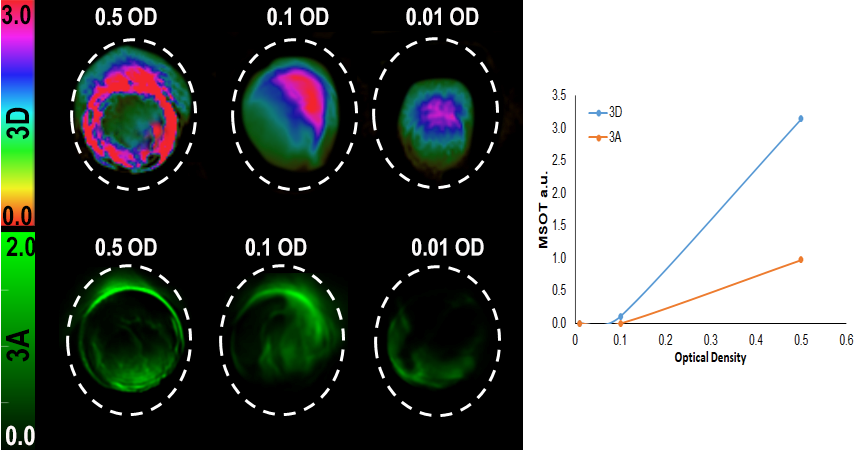


Figure 2S. Varying the concentrations of **3A** and **3D** using MSOT


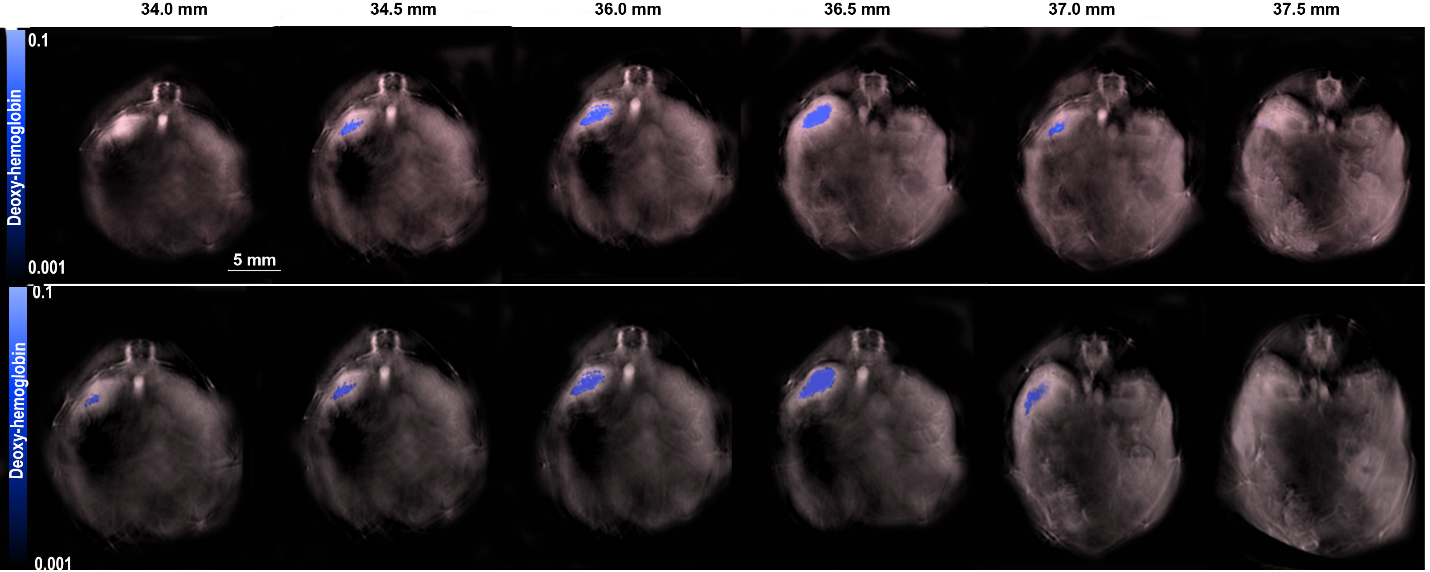
Figure 3S. Showing deoxy-hemoglobin of mice for Figure 5A


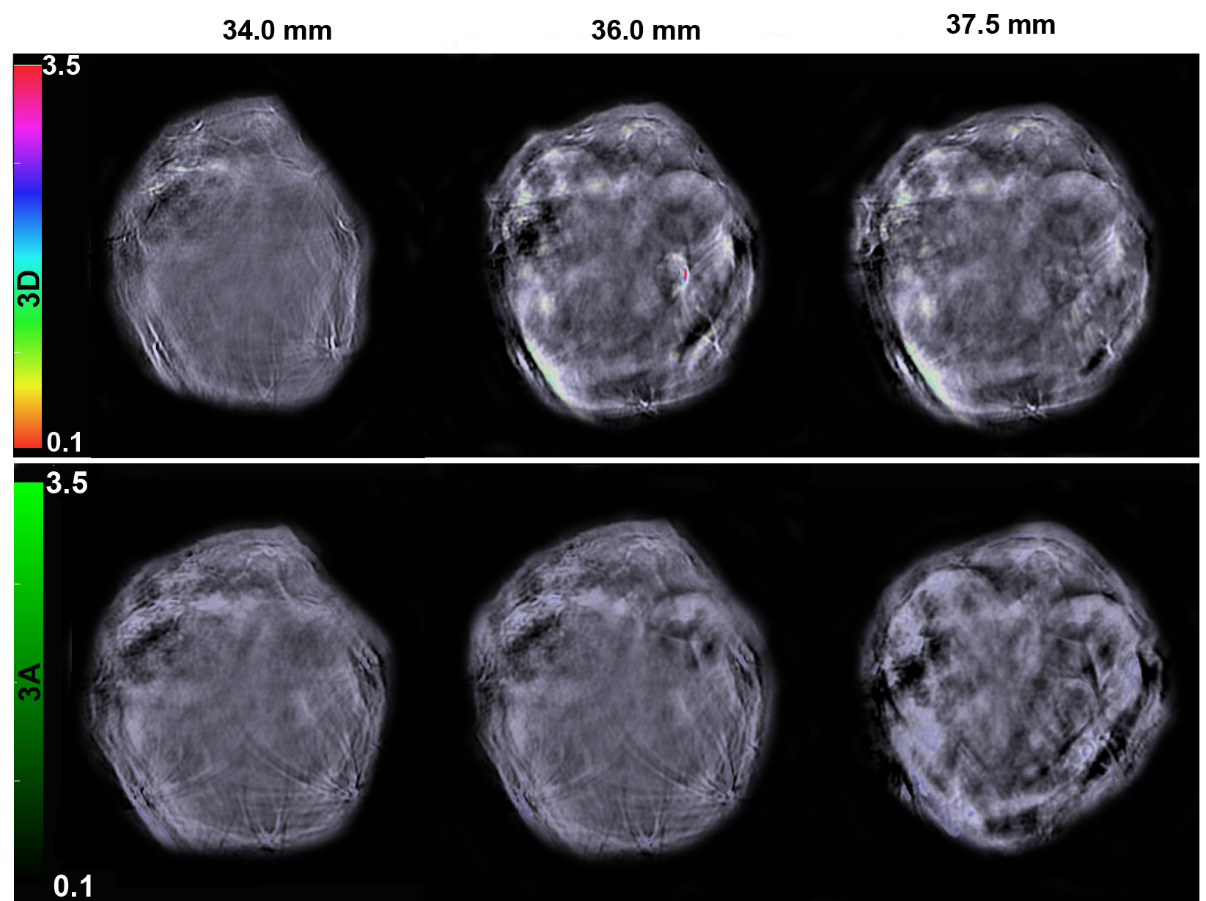


Figure 4S. Showing MOST images in mice injected with silica nanoparticle containing either **3A** or **3D** with no V7 targeting (control).
